# Probiotic Supplementation in Preterm: Feeding Intolerance and Hospital Cost

**DOI:** 10.3390/nu9090965

**Published:** 2017-08-31

**Authors:** Flavia Indrio, Giuseppe Riezzo, Silvio Tafuri, Maria Ficarella, Barbara Carlucci, Massimo Bisceglia, Lorenzo Polimeno, Ruggiero Francavilla

**Affiliations:** 1 Department of Pediatric, Aldo Moro University of Bari, Ospedale Pediatrico Giovanni XXIII via Amendola 276, 70125 Bari, Italy; maria.ficarella@hotmail.com (M.F.); rfrancavilla@gmail.com (R.F.); 2 Laboratory of Nutritional Physiopathology, National Institute for Digestive Diseases, Istituto di Ricerca e Cura a Carattere Scientifico (I.R.C.C.S.), Saverio de Bellis, 70013 Castellana Grotte (BA), Italy; griezzo@gmail.com; 3 Department of Biomedical Sciences and Human Oncology, Section of Hygiene, Aldo Moro University of Bari, 70125 Bari, Italy; silvio.tafuri@uniba.it; 4 Division of Neonatology, Ospedale Perrino, 72100 Brindisi, Italy; bcalucci@lbero.it; 5 Department of Pediatrics, Neonatology Division San Giovanni di Dio Hospital, 88900 Crotone, Italy; m.bisceglia@tin.it; 6 Department of Organ Transplantation, Gastroenterology Section, University Aldo Moro Bari, 70125 Bari, Italy; lorenzo.polimeno@uniba.it

**Keywords:** preterm newborn, feeding intolerance, probiotic

## Abstract

We hypothesized that giving the probiotic strain *Lactobacillus reuteri* (*L. reuteri*) DSM 17938 to preterm, formula-fed infants would prevent an early traumatic intestinal inflammatory insult modulating intestinal cytokine profile and reducing the onset of feeding intolerance. Newborn were randomly allocated during the first 48 h of life to receive either daily probiotic (10^8^ colony forming units (CFUs) of *L. reuteri* DSM 17938) or placebo for one month. All the newborns underwent to gastric ultrasound for the measurement of gastric emptying time. Fecal samples were collected for the evaluation of fecal cytokines. Clinical data on feeding intolerance and weight gain were collected. The costs of hospital stays were calculated. The results showed that the newborns receiving *L. reuteri* DSM 17938 had a significant decrease in the number of days needed to reach full enteral feeding (*p* < 0.01), days of hospital stay (*p* < 0.01), and days of antibiotic treatment (*p* < 0.01). Statistically significant differences were observed in pattern of fecal cytokine profiles. The anti-inflammatory cytokine interleukin (IL)-10, was increased in newborns receiving *L. reuteri* DSM 17938. Pro-inflammatory cytokines: IL-17, IL-8, and tumor necrosis factor (TNF)-alpha levels were increased in newborns given placebo. Differences in the gastric emptying and fasting antral area (FAA) were also observed. Our study demonstrates an effective role for *L. reuteri* DSM 17938 supplementation in preventing feeding intolerance and improving gut motor and immune function development in bottle-fed stable preterm newborns. Another benefit from the use of probiotics is the reducing cost for the Health Care service.

## 1. Introduction

During the third trimester of pregnancy and in the first days after birth, important processes of intestinal maturation take place. Although anatomical differentiation of the human gut is usually achieved within 20 weeks of gestation, the functional maturation of the gastrointestinal tract occurs later and requires organized peristalsis and coordinated sucking and swallowing reflexes that are not established until 29–30 weeks and 32–34 weeks of gestations, respectively [1]. Sensory-motor gastrointestinal functions are strictly related to the infant’s immune system, which plays a crucial role in modulating appropriate and non-exaggerated responses to luminal antigens. This fundamental enteric function, known as “oral tolerance” is based on the interaction between the luminal content (microbiota, food antigens, and other molecules), the intestinal epithelium, and the tolerogenic dendritic cells (DCs) from mesenteric lymph nodes of the gut associated lymphoid tissue (GALT) [2], and is associated with specific cytokine patterns. It has been suggested that the early composition of the intestinal microbiota at birth can influence the correct ontogenesis of the gut barrier, and motor and immune function through a complex neuroendocrine cross-talk [3,4]. Consequences of prematurity like antibiotic usage, feeding type, and being located in neonatal intensive care unit (NICU) may cause an intestinal dysbiosis that affects the intestinal integrity and disrupts the delicate balance between intestinal microbiota and the immune system of premature infants. An aberrant microbial colonization pattern might contribute to the development of an early traumatic inflammatory insult on the gut-brain axis with short- and long-term consequences on gastrointestinal well-being [5].

Early colonization of the gastrointestinal tract with a probiotic may contribute to the neonatal tolerance, as breast-feeding does, resulting in correct gut ontogenesis [6,7,8,9]. Besides, it has been demonstrated that *L. reuteri* DSM 17938 significantly reduced intestinal mucosal levels of IL-8 and interferon (IFN)-γ when newborn rat pups were fed formula containing lipopolysaccharide (LPS) ± *L. reuteri*. *L. reuteri* DSM 17938 was able to significantly reduce the intestinal histological damage produced by LPS plus cow milk formula in the same animal model. Even cow milk formula feeding without LPS produced a mild gut inflammation, evidenced by elevated mucosal IFN-γ and IL-13 levels, and that process could be suppressed by the strain 17938 [10].

In this framework, we hypothesized that giving the probiotic strain *L. reuteri* DSM 17938 to preterm, formula-fed infants would prevent an early traumatic intestinal inflammatory insult modulating intestinal cytokine profile and would reduce the onset of feeding intolerance acting also on gastrointestinal motility.

## 2. Methods

A randomized, double-blinded, clinical, and placebo-controlled trial, was conducted in two Italian neonatal intensive care units (NICUs), Brindisi and Crotone, from January 2011 to November 2012. Preterm neonates (gestational age <37 weeks), within the first 48 h of life: adequate for gestational age weight, formula fed, Apgar score at 5 min >7, <24 h of age, hemodynamically stable, with absence of congenital malformations, inborn errors of metabolism, proven sepsis, or infections at birth were included. The ethical committees of each participating institution approved the study protocol. All preterm newborns were screened at birth and the parents of eligible newborns gave signed consent. Trial registered ClinicalTrials.gov (National Institutes of Health) with the number NCT00985816.

### 2.1. Randomization and Interventions

Preterm newborns were randomly assigned to receive a *L. reuteri* DSM 17938 supplementation or placebo by the use of a computer-generated randomization scheme. The study personnel, health care workers, and parents were masked to the study group allocation. The active study product consisted of freeze-dried *L. reuteri* DSM 17938 suspended in a mixture of pharmaceutical grade sunflower and medium-chain triglyceride oils supplied in a dark bottle fitted with a dropper cap and an individual number indicating the randomization sequence. Five drops of the formulation, delivering a dose of 1 × 10^8^ colony-forming units (CFUs) of *L. reuteri* DSM 17938, were administered to infants in the probiotic group each day regardless of whether enteric feeds were started and until 30 days of life. The placebo consisted of an identical formulation of oils supplied in an identical bottle and was administered following the same protocol as that described for the probiotic group. There were no differences in smell or taste between the two formulations. Analysis of total Lactobacillus counts was performed in our laboratory on three randomly selected bottles from separate batches to ensure the viability of the live bacteria. Each bottle of 10 mL, containing at least 45 dosages, was confirmed to conform with the stated content of *L. reuteri* of at least 4.5 × 10^9^. The study products were stored refrigerated, which keeps the live content at a stable level.

*L. reuteri* DSM 17938 has the ability to colonize the entire human gastrointestinal tract with a blood safety profile similar to *L. reuteri* ATCC 55730 (*L. reuteri* DSM 17938 is daughter strain of *L. reuteri ATCC 55730*). Its colonization is only temporary, and genome annotation did not reveal any further gene or gene cluster known to be involved in virulence or antibiotic resistance [11].

Both the *L. reuteri* DSM 17938 and placebo were manufactured and donated by BioGaia AB (Stockholm, Sweden). The viability and purity of the packaging data was guaranteed by the certificate of analysis provided from the manufacturer for each of the batches. In order to minimize differences in feeding and nutrition practices among participating NICUs, the preterm newborns were all exclusively bottle-fed with the same preterm standard formula. Minimal Enteral Feeding was started on day 1 or 2 with small amounts of enteral feedings of formula at intakes of 10–20 mL/kg/day. The limit of tolerance for increasing formula amount (10–20 mL/kg/day) was set at 50% of previous feeds on the assessment of pre-feed gastric residual volumes. Parenteral nutrition, when needed, was started on the second day of life following a standardized protocol.

### 2.2. Symptoms and Data Evaluation

Anthropometrical parameters, occurrence of adverse reactions, time to regain birth weight, time taken to reach full enteral feeding, duration of antibiotic treatment, days of hospital stay, and stool frequency were recorded. In order to perform quantification of fecal cytokines and fecal calprotectin, a stool sample was obtained at the end of supplementation (30 days of life). Fecal interleukin (IL)-1β, IL-6, IL-8, IL-10, IL-17, TNFα, and fecal calprotectin were evaluated. Briefly, for measurement of fecal IL, TNF, and calprotectin, fecal samples were taken from the diaper with a sterile plastic spoon. Samples were stored in a sterile screw cap tube at 2–8 °C for a maximum of 7 days. Samples were extracted and diluted 1:50 with an incubation buffer. The homogenate was microcentrifuged for 5 min at 10,000× *g*, and the supernatant was stored at −20 °C until analysis. The concentrations of fecal cytokines were determined in each fecal sample photometrically with commercially available enzyme-linked immunosorbent assay (ELISA) kits (IL-1β, IL-6, IL-8, IL-10, IL-17, and TNFα (IDK^®^ TNFα), ELISA (Immunodiagnostik, Bensheim, Germany), according to the instructions provided by the manufacturer. Calprotectin levels were measured by ELISA using the Calprotectin-MPR8/14 kit (Buhlmann, Basel, Switzerland). All of the concentrations quantified were adjusted to the wet weight of the individual fecal sample.

Scintigraphy is considered the “gold standard” among methods to evaluate gastric emptying in clinical practice [12]. Scintigraphy allows a complete and physiologic study of the motor function of the stomach. However, it is an expensive technique and specialized personnel from nuclear medicine are needed to perform a scintigraphic study. Moreover, scintigraphy induces significant radiation emission [13], and as result it has limited application in newborns and children, and does not allow repetitive measurements in a short period of time. In comparison, the study of gastric emptying time by ultrasound is a non-invasive method and can be used for research or practical purposes to evaluate gastric emptying in healthy adults, children, and newborns, and in those with gastrointestinal motility disorders. On considering these factors, an ultrasound gastric emptying examination was performed on day 30 after birth, according to a previously reported procedure [14,15]. Lastly, a cost-analysis assessment during NICU hospitalization was performed.

### 2.3. Outcome Measures

The primary outcome was the assessment of *L. reuteri* DSM 17938 supplementation on feeding tolerance through evaluation of cytokine fecal profile, clinical parameters, and ultrasound measurement during the first month of life. The secondary outcome was to evaluate the costs of supplementation, calculated based on duration of hospitalization.

### 2.4. Sample Size

To calculate the minimum sample size, we used previous published data [7] about the reduction of average time of gastric emptying; we hypothesized a reduction of 10% of the average time and we used an alpha of 0.05 and a power of 0.9. Using Stata Software, we estimated a required sample size of 20 for each group.

We repeated the sample size calculation using, as the main outcome, the reduction of the cytokine fecal profile and setting an alpha of 0.05 and a power of 0.9. In this case, the required sample was around 30. We hypothesized a drop-out rate of 20%; then, we planned to enroll 72 subjects.

### 2.5. Data Analysis

Clinical and functional gastrointestinal variables were considered prospectively. The mean and standard deviation of each quantitative parameter were calculated and means were compared by unpaired Student’s *t*-test, after establishing the normal distribution of variables by Bartlett’s test. Significance was set at *p* < 0.05. To evaluate the costs of the hospitalizations, we used the Diagnosis Related Group (DRG) rate as reported on the Official website of Italian Ministry of Health for DRG 388 (prematurity without major risks).

## 3. Results

A total of 72 preterm newborns were eligible. Twelve newborns were excluded from analysis because of parental refusal to participate (three neonates), withdrawal from the study (five neonates), or maternal desire to breastfeed (three neonates). The need for exclusive bottle-feeding should be considered the most important covert/clearly expressed cause of high drop-out of these infants. However, the response rate was 83.3% (60/72), higher than that expected (80%). A total of 60 preterm newborns were randomly assigned to *L. reuteri* DSM 17938 or to the placebo group. Clinical and demographic characteristics of the infants at birth are shown in Table 1. No significant differences were observed between the two groups at baseline. The newborns receiving *L. reuteri* DSM 17938 had a significant decrease in the number of days needed to reach full enteral feeding (*p* < 0.01), days of hospital stay (*p* < 0.01), and days of antibiotic treatment (*p* < 0.01) compared with those given placebo. Further, significant differences were found in time to regain birth weight, which was reduced in the *L. reuteri* DSM 17938 supplemented group (*p* < 0.05), and in body weight at the end of the study, which was lower in the placebo group (*p* < 0.05), Table 2. Multiple statistically significant differences were observed in the pattern of fecal cytokine profiles between the two groups. The anti-inflammatory cytokine IL-10, which is involved in immune tolerance, was increased in newborns receiving *L. reuteri* DSM 17938. Pro-inflammatory cytokines: IL-17, IL-8, and TNFα levels were increased in newborns given placebo. The newborns receiving *L. reuteri* DSM 17938 had a significant decrease in calprotectin level compared with the placebo group. The IL-1β was increased in the group treated with *L. reuteri* DSM 17938. IL-6 was increased in newborns receiving *L. reuteri* DSM 17938, but the difference was not statistically significant (Table 3). Regarding gastric emptying, significant differences in the half-emptying (T1/2) time and fasting antral area (FAA) were reported, confirming the improved motility pattern in the probiotic administration group (Table 4). There were no adverse events related to the trial in the *L. reuteri* DSM 17938 supplemented babies.

The cost of treatment saved by the reduction of hospitalization by giving *L. reuteri* DSM 17938 amounted to 2043 Euros per infant.

## 4. Discussion

*L. reuteri* DSM 17938 supplementation in preterm newborns improves intestinal motility and changes the cytokine profile in stools. Our study underlines the potential beneficial effects of *L. reuteri* DSM 17938 supplementation on clinical and functional variables related to maturation of gastrointestinal function. In particular, it shows that oral supplementation with *L. reuteri* DSM 17938 improves feeding tolerance in preterm newborns with clinical effects on growth, hospitalization, and antibiotic treatment.

Primary colonization of the gut can be considered an important step in the development of intestinal functions and the transferal of the microbiota at birth from maternal vaginal and intestinal flora to the newborn gut is fundamental [16]. The continuous and complex cross-talk between the gut and its microbial content is a normal part of development and plays a crucial role in the ability to distinguish harmless bacterial and food antigens from potentially dangerous antigens. This function requires a sophisticated system that is responsive to a wide variety of microbial and food antigens that transit or populate the gut [17].

An imbalance of normal intestinal microbiota, or the host response to such an imbalance are thought to be involved in the pathogenesis of several intestinal diseases [18]. The beneficial effect of probiotic supplementation on feeding intolerance and immunomodulation has been reported in several studies [4]. A recent study by Rojas [19] reported feeding intolerance episodes and duration of hospitalization significantly lower in preterm infants <1500 g exposed to *L. reuteri* DSM 17938 in comparison to placebo exposed infants. Different from the study by Rojas, our newborns supplemented with probiotic reached the full enteral feeding in a shorter time.

The specific mechanism of probiotic supplementation on gastrointestinal function is not yet clear. Functional components of the human gastrointestinal tract do not evolve simultaneously and it has been shown that a reduced or an abnormal microbial colonization during the first months of life would provoke a slower postnatal maturation of epithelial cell barrier functions, of neuronal route, and of the immunomodulation system of the GALT [20]. This aberrant development of gut functions could finally lead to mucosal inflammation and play a pivotal role in the development of feeding intolerance [21] or other diseases later in life. In an animal model, *L. reuteri* DSM 17938 significantly reduced intestinal mucosal IL and interferon levels, and such reduction corresponded to a reduction in the low grade histological damage induced by cow milk formula feeding [10].

In agreement with these data, in our study, early probiotic supplementation induced a decrease in fecal pro-inflammatory cytokines, IL-17, IL-8, and TNFα, and an increase in the fecal anti-inflammatory cytokine IL-10. Our data suggest a potential anti-inflammatory effect of probiotic with a shift in the tolerogenic mechanism on naïve CD4+ T cells that suppress the expression of T effector cells (Th1 and Th2) and stimulate the expansion of regulatory T cells (Tregs). Furthermore, significant changes in IL-1β and IL-6 levels were found in preterms given probiotic compared to placebo, and previous studies in animals have shown that these two cytokines can have excitatory and neuromodulatory roles in the myenteric plexus, stimulating gastrointestinal motility [22,23]. Also, fecal calprotectin, a well-known marker of gut inflammation, was reduced in the infants supplemented with *L. reuteri* DSM 17938. The modulation of fecal calprotectin by this specific probiotic strain has been reported in the literature [24,25].

Another important aspect is the growing evidence suggesting that the intestinal microbiota may be emitting and receiving a multiplicity of signals to and from the brain, thus playing a critical role in the modulation of the gut-brain axis [26,27]. An immature ontogenesis of this bidirectional interrelationship between the enteric microbiota and the nervous system could affect the pathophysiology of feeding intolerance [28]. In this context, the strength of contemporary action on the motility and immunity of the intestine could result in a better functionality of the whole intestinal function. Our study demonstrates that gastric motility was improved in preterm infants given *L. reuteri* DSM 17398, as shown by the significantly increased gastric emptying time, and the significantly reduced FAA. The clinical counterpart of such improved gastric activity is the decreased gastric residual in preterm infants given *L. reuteri* DSM 17938 and the consequent earlier achievement of full enteral feeding and the faster regain to birth weight compared to the placebo group. This bacterial strain has already been used in a pediatric population [29], and a recent paper [30] showed that LR DSM 17938 increased both colonic migrating motor complex frequency and velocity in an animal model. The authors, based upon the effects of LR DSM 17938 on the adult mouse colon, speculated that this approach may help to screen and identify the therapeutic effect of LR DSM 17938 on constipation and, generally, to correlate the given effect of the probiotic on the enteric nervous system with the action on GI motility.

## 5. Conclusions

Our study demonstrates an effective role for *L. reuteri* DSM 17938 supplementation in preventing feeding intolerance and improving gut motor and immune function development in bottle-fed stable preterm newborns. The physiological mechanisms underlying these effects may involve changes in cytokine inflammatory patterns. Finally, in light of our cost-analysis assessment, another benefit from the use of this probiotic is in the form of reductions in the costs for the Health Care service.

## Figures and Tables

**Table 1:** Clinical and demographic data at baseline.

Total *n* = 60	*L. reuteri* DSM 17938 (*n* = 30)	Placebo (*n* = 30)	*p*
Gestational age	30.2 ± 1.2	30.1 ± 1.2	n.s.
Gender (M/F)	15/15	16/14	n.s.
Delivery (VD/CD)	4/26	5/25	n.s.
Birth weight (g)	1471.5 ± 455.1	1406.6 ± 536.4	n.s.

Gender: M/F = Male/Female; Delivery: vaginal delivery (VD)/cesarean delivery (CD). Non significant (n.s.)

**Table 2:** Clinical results at the end of the study.

Clinical Parameter	*L. reuteri* DSM 17938	Placebo	*p*
Time taken to reach full enteral feeding (day)	4.2 ± 1.1	7.5 ± 3.2	<0.01
Day of hospitalization (day)	13.4 ± 2.2	22.4 ± 3.2	<0.01
Duration of antibiotic treatment (day)	4.2 ± 4.3	12.5 ± 7.2	<0.01
Time to regain birth weight (day)	6.4 ± 1.6	7.3 ± 1.3	<0.05
Weight at the end of the study (g)	1955.3 ± 653.4	1737.6 ± 512	<0.05
Stool frequency (*n*/day on the last week)	2.5 ± 0.7	2.8 ± 0.9	<0.05

**Table 3:** Fecal cytokines.

Group	IL-1β	IL-8	IL-10	IL-17	Calprotectin	TNFα	IL-6
	pg/mL	pg/mL	pg/mL	pg/mL	μg/g	pg/mL	pg/mL
LR	57.4 ± 73.3	56.7 ± 72.4	6.3 ± 3.2	6.5 ± 1.9	246.6 ± 78.4	8.0 ± 3.1	3.2 ± 2.8
Placebo	17.1 ± 16.7	197.3 ± 222.1	4.2 ± 1.7	8.8 ± 3.5	323.9 ± 111.7	12.7 ± 7.7	2.9 ± 1.7
*p*	0.04	0.04	0.02	0.02	0.01	0.01	n.s.

LR = *L. reuteri* DSM 17938; IL = interleukin; TNF = tumor necrosis factor.

**Table 4:** Gastric emptying parameters at the end of the study.

Parameter	*L. reuteri* DSM 17938	Placebo	*p*
T1/2 (Half-emptying time) (min)	73.8 ± 7.5	80.4. ± 6.1	0.0004
Fasting antral area (cm^2^)	0.6 ± 0.2	0.8 ± 0.3	0.009

## Abbreviations

DCs	Dendritic cells
GALT	Gut associated lymphoid tissue
NICU	Neonatal intensive care unit
CFU	Colony forming unit
IL	Interleukin
